# Hello and goodbye Research and Practice in Thrombosis and Haemostasis

**DOI:** 10.1016/j.rpth.2024.102353

**Published:** 2024-02-15

**Authors:** Mike Makris, Mary Cushman

**Affiliations:** 1 Department of Infection, Immunity and Cardiovascular Disease, University of Sheffield, Sheffield, UK; 2 Department of Medicine, Larner College of Medicine at the University of Vermont, Burlington, Vermont, USA

2017 saw the launch of the Research and Practice in Thrombosis and Haemostasis (RPTH) journal with M.C. as its founding editor. RPTH is the online sister journal to Journal of Thrombosis and Haemostasis (JTH), which together constitute the official journals of the International Society of Thrombosis and Haemostasis (ISTH). 2024 sees the first change in editor-in-chief with M.M. taking the reins. The changeover has been more complex than usual since it coincided with a change in publisher, from Wiley to Elsevier, and then in our manuscript management system, from ScholarOne to Editorial Manager. We have worked together over several months to make the transition as smooth as possible for our authors, reviewers, readers, and staff.

RPTH is an open access journal where the authors pay an article processing charge (APC) rather than relying on subscription access. We believe this is the future model for most scientific publications, as funders of research increasingly insist that the research they fund should be accessible to everybody as soon as accepted for publication. Accessibility to all is also a critical advantage over firewalls, which prevent access; this issue is particularly important in low- and middle-income countries and also to those at institutions that can no longer afford soaring subscription fees.

We are aware of the major competition from the increasing number of journals, both conventional and predatory, publishing in thrombosis and hemostasis. We are grateful for the support we receive from our community, reflected by rapidly increasing number of researchers wishing to publish with us, the increasing quality of submissions, and the increasing readership based on the number of downloads of papers from the journal website. We are proud that despite growing submissions, our time to first decision has remained stable at 15.2 days, which reflects our amazing team of associate editors and those who review for us.

As a newer journal, and by virtue of being online only, we have been able to innovate, and this will continue. A popular novel feature of RPTH has been the Illustrated Review articles, which were introduced by M.C. and Alisa Wolberg and are now supervised by an illustrated materials associate editor, Michelle Sholzberg [1]. These review articles are scholarly reviews of the best quality possible. The first illustrated review on polyphosphate by Catherine Baker and colleagues [2] received a large readership, huge attention on social media, and has been cited 56 times. Each year, illustrated reviews tend to be our top downloaded and cited articles. Other journals are adopting the format, which is exciting! We also publish State-of-the-Art capsule articles that quickly and clearly summarize a conference and are also popular with our readers [3,4]. Both of these novel formats for publishing will continue with the new editorial team.

It is important that publications are disseminated and discoverable as much as possible. One of the strengths of RPTH is its social media presence especially on X, formerly known as Twitter. This presence is associated with greater citation impact and allows easy access to followers of the latest science. We are ably assisted by 2 social media associate editors. Coinciding with the change in editor-in-chief, Ismail Raslan and Erica Sparkenbaugh have taken over this role from Megan Brown and Yazan Abou-Ismail. We work with authors of accepted manuscripts to make their papers more visible through search engine optimization and social media exposure.

Diversity, equity, and inclusion (DEI) have been central to the development of the journal from the start, with the target being the reflection of the ISTH membership in all aspects of the journal. Forty-eight percent of the ISTH membership identify as female and this has been the target for authors, invited reviewers, associate editors, editorial board members, and invited review authors. This target is close to being achieved for gender equality, but geographical and race equality are more challenging, partly due to much of the hemostasis and thrombosis research being concentrated in Europe and North America. The overall DEI objectives passionately led by M.C. will continue to be the aims of the new editorial team. It is important that DEI data are regularly collected and presented to the scientific community both as publications and online articles [5]. We are happy to report that starting in 2024, our ISTH sister journal, Journal of Thrombosis and Haemostasis, will also commence collecting and presenting their DEI data, as will the ISTH society overall.

As one of our associate editors, Yotis Senis recently pointed out, a scientific journal is like a jumbo jet with the editor-in-chief being the pilot. Without the crew and other support staff, you do not get very far. The copilot in this journey has been the deputy editor Cihan Ay who will continue until the transition is fully completed, before stepping down. We could not have done our job without the outstanding help from our brilliant team of associate editors. Pantep Angchaisuksiri, Yotis Senis, Cihan Ay, and Suzanne Cannegieter are standing down and Nick van Es, Carsten Deppermann, and Luuk Scheres are our newest associated editors. Lana Castellucci, Johnny Mahlangu, Vania Morelli, Suely Rezende, Bethany Samuelson Bannow, Kristen Sanfilippo, Henri Spronk, Neil Zakai, and Michelle Sholzberg will continue in their associate editor roles.

As a new era begins for the journal, we continue to look forward to your submissions and to work with you to enhance the visibility of your research and the field of thrombosis and hemostasis in general.
